# Parasympathetic nervous activity and CTRA gene expression among healthy young adults in Japan

**DOI:** 10.1016/j.bbih.2025.101040

**Published:** 2025-06-19

**Authors:** Yoshino Murakami, Takeshi Hashimoto, Steve Cole

**Affiliations:** a Faculty of Sport and Health Science, Ritsumeikan University, Kusatsu, Shiga, Japan; b Department of Medicine and Psychiatry and Biobehavioral Sciences, UCLA School of Medicine, Los Angeles, CA, USA

**Keywords:** Autonomic nervous activity, Neuroendocrine system, Inflammation, Japan, Cultural difference

## Abstract

Previous research suggests that parasympathetic nervous system (PNS) activity may inhibit the leukocyte Conserved Transcriptional Response to Adversity (CTRA) which has been observed in individuals exposed to prolonged stressors like loneliness, social isolation, and bereavement. Previous PNS-CTRA studies have focused on Western populations, raising questions about the generalizability of these findings across different cultural and ethnic backgrounds. This study examined the relationship between PNS activity (as indexed by heart rate variability; HRV) and CTRA gene expression in young, healthy adults in Japan (n = 26; Mean age = 26; 34.6 % female). In analyses that controlled for demographic and behavioral covariates (including age, sex, ethnicity, smoking, alcohol, and BMI), results showed a significant inverse relationship between HRV and CTRA gene expression (i.e., lower expression of pro-inflammatory genes and elevated expression of Type I interferon response genes). Convergent validation analyses of genome-wide transcription factor activity linked HRV to up-regulation of Interferon Response Factors and down-regulation of NF-κB. These results parallel previous findings from Western samples, confirming that PNS neuro-immune regulation generalizes to an population based in Japan, as part of broader East Asian region and identifying HRV as a useful index for optimizing immune health in diverse populations.

## Highlights

• Higher PNS is linked to reduced inflammation including CTRA in western populations.
• Race and ethnicity may modify the relation between PNS and inflammatory biomarkers.
• Higher PNS was also related to lower CTRA gene expression in Japanese young adults.
• Neuro-immune regulation of the CTRA appeared similar in Asian vs Western populations.

## 1 Introduction

The sympathetic nervous system (SNS) and parasympathetic nervous system (PNS), interactively affect various physiological systems, including the immune system (Kenney and Ganta, 2014). A series of RNA profiling studies found that human beings who were exposed to various adverse environmental conditions (e.g., social isolation, loneliness, bereavement, psychological stress) for extended periods of time showed a recurrent pattern of differences in immune cell gene expression profile named as Conserved Transcriptional Response to Adversity (CTRA) (Cole, 2014). CTRA is characterized by upregulation of genes involved in inflammation (e.g., *IL1B, IL6, IL8/CXCL8, COX2/PTGS2, and TNF*) and downregulation of genes involved in Type I interferon-mediated innate antiviral responses (e.g., *IFI-, MX-, and OAS-family genes*) (Cole, 2019). Extended exposure to adverse environmental conditions can activate the SNS and trigger “fight-or-flight stress responses” (Hu et al., 2022) mediated by the neurotransmitter, norepinephrine. These responses are transduced by leukocyte beta-adrenergic receptors and result in up-regulation of pro-inflammatory genes via activation of the NF-κB/Rel transcription factor family and decreased expression of Type I interferon response genes via inhibition of Interferon Response Factors (IRF) (Cole, 2019).

While the role of SNS in activating CTRA profiles has been repeatedly demonstrated, much less is known about the possibility that PNS activity might reciprocally inhibit CTRA gene expression (i.e., down-regulate inflammation and stimulate Type I interferon antiviral genes). PNS activity can be indexed by heart rate variability (HRV; i.e. variation in the time intervals between successive heart beats that results from respiration-induced fluctuations in vagal nerve activity, which acts to increase in inter-beat intervals) (Shaffer and Ginsberg, 2017), and augmented PNS activity is highly correlated with increase in HRV (Hayano et al., 1991). Although a small number of studies have examined associations between HRV and CTRA profiles (Rahal et al., 2021, Ricon-Becker et al., 2021; Sloan and Cole, 2021), these studies have been conducted on Western samples, and the generality of these findings across age groups, ethnic groups, and cultural contexts remains unclear. Sloan & Cole found that HRV was associated with reduced activity of the pro-inflammatory NF-κB transcription control pathway and increased activity of the antiviral IRF transcription control pathway in participants of the Midlife in the US (MIDUS) (n = 863, M_age_ = 50, SD = 13). Rahal et al. similarly observed the relation of higher HRV with higher Type I interferon response gene expression, consistent with down-regulation of CTRA in a young population from local high schools in Los Angeles (n = 90, M_age_ = 16, SD = 0). Rahal et al. demonstrated positive links between high-frequency (HF) HRV (0.15Hz–0.40Hz) and Type I interferon response gene expression (B = 0.12, SE = 0.02), as well as a negative relation between HRV and CTRA (B = −0.06, SE = 0.02) after adjusting for ethnicity. Of note, however, the relationship between HRV and gene expression profiles may vary as a function of ancestry or ethnic background. In fact, Rahal et al. discussed the possibility of cofounding effects related to ancestry-associated genetic factors influencing the association between HRV and CTRA within their paper.

Given that existing studies linking PNS activity to CTRA gene expression have taken place solely in Western countries (with the vast majority of samples coming from the United States), but existing data suggest potential racial/ethnic variation in these effects, we sought to expand the current evidence base to encompass Japanese adults and thereby broaden our understanding of the association between HRV and CTRA.

### 1.1 Purpose

Based on these backgrounds, this study aimed to investigate the relationship between HRV and CTRA in a population of young, healthy individuals residing in Japan.

### 1.2 Hypothesis

Based on prevision research linking PNS activity to reduced CTRA gene expression (Ricon-Becker et al., 2021; Rahal et al., 2021; Sloan and Cole, 2021), as well as the broader psychological research literature linking general psychological well-being to reduced CTRA gene expression ( Fredrickson et al., 2015, Snodgrass et al., 2022), including among East Asian populations, (e.g., Kitayama et al., 2016; Lee et al., 2020), we hypothesize that, similarly to the patterns suggested so far, HRV in residents of Japan will also demonstrate a negative correlation with CTRA gene expression.

## 2 Methods

### 2.1 Participants

Healthy young individuals (20–39 years) were recruited through posters advertisements and social media.

### 2.2 Exclusion criteria

Individuals who meet any of criteria below were excluded from participation candidates.

1. Individuals those under 20 or over 40 years old.
2. Individuals currently diagnosed with any of the following conditions by a physician: addiction, depression, anxiety disorder, eating disorder, bipolar disorder, epilepsy, schizophrenia, or dementia.
3. Individuals suffering from illnesses that require regular medical visits.
4. Individuals regularly taking prescribed medications or psychotropic drugs for the purpose of mental stability.
5. For female, those experiencing irregular or absent menstruation.

### 2.3 Procedures

Participants visited the laboratory at Ritsumeikan University, Biwako-Kusatsu in the morning (between 8:00AM–11:30AM) for a single day between June and September 2022. Participants were informed about the study and provided informed consent before the proceeding. The data correction involved questionnaire survey (demographic characteristics) and physiological measurements (height, weight, Body Mass Index [BMI], ECG recording and blood sampling). All the physiological measurements were completed in the morning (before 12:00 p.m.). For female participants, we targeted individuals with stable menstrual cycles, confirmed their menstrual start date for the past three months, and scheduled the experiments during the premenstrual phase (defined as luteal phase). To control for the effects of other lifestyle factors on the results, participants were instructed to refrain from alcohol consumption, intense exercise and caffein intake from the day before the experiment. They were also instructed to ensure sufficient sleep, have breakfast at least 3 h before the experiment, and avoid excessive physical activity when commuting to the laboratory using bus or other transportation. Laboratory environment was carefully maintained as constant as possible (room temperature [°C] 23 ± 2.6, humidity [%] 64 ± 7.6). HRV was measured by using an electrocardiogram (ECG) at rest in the supine position for 10-min. The R waves from the ECG recording were checked using the LabChart. The HRV during the last 5-min was analyzed in the time domain (root mean square of successive differences [RMSSD] in ms, and standard deviation of NN intervals [SDNN] in ms).

RMSSD is marker of vagal function which strongly correlate with HF (Task Force of the European Society of Cardiology and the North American Society of Pacing and Electrophysiology, 1996). SDNN correlates with total power (both sympathetic and parasympathetic activity) (Task Force of the European Society of Cardiology and the North American Society of Pacing and Electrophysiology, 1996).

The study was conducted after obtaining approval from the Institutional Review Board (IRB) of Ritsumeikan University (IRB Approval Number: BKC-LSMH-2021-056-2).

### 2.4 Blood sampling

Following the ECG recording, participants kept a supine position, and venous blood (2.5 ml) was collected by antecubital venipuncture into PAXgene RNA tubes and stored at – 80 °C. Tubes were subsequently shipped in batch to the UCLA Social Genomics Core Laboratory for assay and analysis as previously described (Cole et al., 2020). Briefly, RNA was extracted (Qiagen RNeasy), tested for suitable mass (RiboGreen RNA) and integrity (Agilent TapeStation), converted to cDNA using a high-efficiency mRNA targeted reverse transcription system (Lexogen QuantSeq 3′ FWD), and sequenced on an Illumina NextSeq instrument by Lexogen Services GmbH, all following the manufacturer's standard protocols. Sequencing targeted 5 million 100-nt single stranded sequencing reads per sample (achieved mean = 5.3 million), each of which was mapped to the GRCh38 reference human transcriptome using the STAR aligner and quantified as gene transcripts per million (TPM) total mapped sequencing reads. TPM values were floored at 1 to suppress spurious variability, and log_2_ transformed to stabilize variance, and z-score standardized within gene for statistical model analyses as outlined below.

### 2.5 Statistical analysis

Following a standard statistical analysis of CTRA gene expression used in previous research (Cole et al., 2015), we used a mixed effect linear model to quantify the association between HRV measures (IBI SDNN and RMSSD) and average expression of 53 pre-specified CTRA indicator gene transcripts, including 19 pro-inflammatory genes (*IL1B, IL8, TNF, PTGS1, PTGS2, FOS, FOSL2, JUN, JUNB, JUND, NFKB1, NFKB2, REL, RELA*, and *RELB*) and 34 genes involved in type 1 interferon responses (transcripts negatively weighted to reflect their inverse contribution to the CTRA profile: *GBP1, IFI27, IFI27L1-2, IFI30, IFI35, IFI44, IFI44L, IFI6, IFIH1, IFIT1-3, IFIT5, IFIT1B, IFITM1-3, IRF2, IRF7-8, MX1-2, OAS1-3, OASL*, and *JCHAIN*). To suppress the effects of spurious measurement variability, 7 genes with no detectable RNA expression or minimal variation (SD < 0.5 log_2_ TPM) were removed from the final analytic subset (*FOSL1, IFITM5, IFNB1, IGLL1, IGLL3P, IL1A, IL6*), leaving 47 analyzed CTRA indicator gene transcripts. Mixed effect linear models (SAS PROC MIXED) quantified the association between average CTRA indicator gene expression (treated as a repeated measure) and HRV measures while controlling for the effects of age, sex, race/ethnicity (Japanese vs Non), BMI, average drinks of alcohol per week over the last month, history of regular smoking, student vs non-student status, marital status, and status as a parent vs non-parent. Analyses also included a random subject-specific intercept to control for correlation among genes. This mixed effect model quantifies the association between HRV and average expression of multiple CTRA indicator transcripts in a single statistical test, so there is no opportunity for multiple hypothesis testing across individual genes to inflate Type I error rates. To account for the effects of multiple testing across the 2 different HRV measures examined in our primary analysis (SDNN and RMSSD) we include false discovery rate (FDR)-adjusted *q*-values alongside individual *p*-values (Benjamini et al., 1995). These analyses did not involve any testing of associations between individual gene transcripts and HRV measures, as the present sample size is not sufficiently large for any well-powered analysis of individual genes (which are subject to greater noise and greater multiple test correction burden than a pre-specified multi-gene composite). However, individual gene associations are provided in Supporting Information File 1 as descriptive results.

To ensure that results from primary analyses were not distinctive to the a priori-specified CTRA indicator gene set, we cross-validated those results using a second well-established genome-wide bioinformatic approach that quantifies differences in the activity of two key a priori-specified transcription control pathways that structure the CTRA gene expression profile: the pro-inflammatory NF-κB/Rel transcription factor family and the antiviral interferon response factor (IRF) family (Cole, 2019). In these analyses, all transcripts (genome-wide) that show ≥2-fold differential expression across the range from a low value of HRV (2 SD below the mean) to a high value of HRV (2 SD above the mean) in the covariate-adjusted analysis specified above served input into TELiS promoter-based bioinformatic analysis (Cole et al., 2005) to quantify the relative abundance of transcription factor-binding DNA motifs (TFBMs) for NF-κB (TRANSFAC V$P50RELAP65_Q5_01) and IRF (V$IRF_Q6) in those genes’ core promoter DNA sequences. Transcriptional activity was quantified by the average (log 2-) ratio of TFBMs in up-vs. down-regulated promoter sequences across nine combinations of three core promoter sequence lengths (−300, −600, and −1000 to + 200 nucleotides relative to the RefSeq transcription start site) and three TFBM detection stringencies (TRANSFAC mat_sim values of 0.80, 0.90, and 0.95). Statistical testing was based on standard errors derived from bootstrap resampling of linear model residual vectors (which controls for correlation among genes within individuals; Efron and Tibshirani, 1994).

## 3 Results

Study sample characteristics are presented in Table 1. 49 participants were enrolled in this study. Of these, 23 were excluded from HRV analysis because of noisy data or due to connectivity issues. This analysis included data from all 26 participants who had valid measures of both HRV and gene expression. Mean age ±SD was 26 ± 5 years. 17 were men and 9 were women. Participants’ BMI was 22.1 ± 3.3 kg/m^2^. Participants primarily identified as Japanese (88.5 %), with smaller percentages identifying with other ethnic background. The distributions of HRV indices (RMSSD and SDNN) were 90.26 ± 31.37 ms and 92.43 ± 40.34 ms, respectively. Regular alcohol consumption was negatively related with both RMSSD and SDNN (vs. RMSSD: *r*_*s*_ = −0.562, *p* < 0.01, vs. SDNN: *r*_*s*_ = −0.415, *p* < 0.05). Age was negatively related with only RMSSD (*r*_*s*_ = −0.401, *p* < 0.05).

**Table 1:** Sample characteristics

A.	Mean	(SD)	$r_{S}^{1}$ (p-value)with SDNN	$r_{S}^{1}$ (p-value)with RMSSD
**Age**	26	(6)	−0.305 (0.130)	**−0.401** (**0.042**)
**Body Mass Index (kg/**$m^{2}$**)**	22.1	(3.3)	−0.185 (0.365)	−0.231 (0.256)
**Sex**	**n**	(**%**)	0.243 (0.233)	0.296 (0.141)
Male	17	(65.4)		
Female	9	(34.6)		
**Race**			−0.056 (0.785)	0.040 (0.846)
Japanese	23	(88.5)		
non-Japanese	3	(11.5)		
**Social status**			−0.113 (0.582)	−0.195 (0.339)
Student	14	(53.8)		
non-Student	12	(46.2)		
**Regular Alcohol consumption**			**−0.415** (**0.035**)	**−0.562** (**0.003**)
<1 time/week	19	(73.1)		
1-2 times/week	4	(15.4)		
more than 3 times/week	3	(11.5)		
**Regular Smoking**			0.093 (0.650)	−0.040 (0.846)
Yes	25	(96.2)		
No	1	(3.8)		
**Marital status**			0.007 (0.975)	−0.046 (0.825)
Yes	5	(19.2)		
No	21	(80.8)		
**Parental status**			−0.056 (0.785)	−0.169 (0.41)
Yes	3	(11.5)		
No	23	(88.5)		

B.	Root mean square of successive differences [RMSSD]	Standard deviationof NN intervals [SDNN]
**HRV mean (SD)**	90.26 (31.37)	92.43 (40.34)

1 Sample (unudjusted) association with HRV (root mean square of successive differences [RMSSD], standard deviation of NN intervals [SDNN]: Spearman's rank correlation coefficient rs point estimate (p-value).

**Fig. 1:**
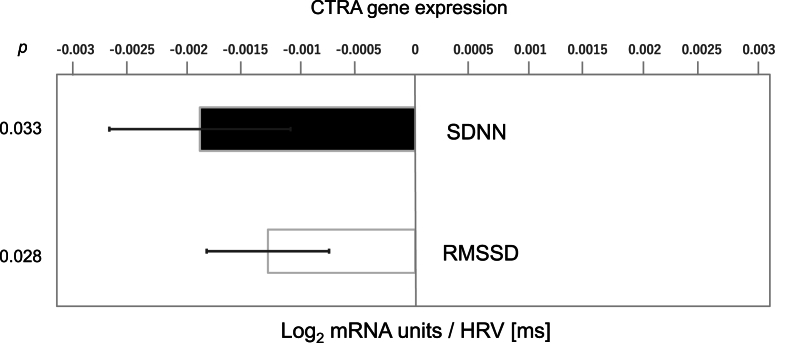
HRV (standard deviation of NN intervals; SDNN and root mean square of successive differences; RMSSD) and CTRA gene expression. Relation of CTRA gene expression to SDNN and RMSSD, while controlling demographic characteristics and health behaviors (smoking, alcohol, BMI).

In primary analyses of CTRA gene expression that controlled for demographic characteristics and health behaviors (smoking, alcohol, BMI), results identified a significant inverse association between CTRA indicator gene expression and two time-domain measures of HRV: SDNN (−0.0019 ± SE 0.0008 log_2_ mRNA units per HRV ms, standardized *b* = −0.145 ± 0.062, *p* = 0.033, FDR corrected *q* = 0.033) and RMSSD (−0.0013 ± 0.0005, *b* = −0.150 ± 0.062, *p* = 0.028, FDR corrected *q* = 0.033) (Fig. 1). These associations correspond to a 32 % lower CTRA gene expression level for study participants with relatively high SDNN HRV values (2 SD above the mean) compared to those with relatively low SDNN HRV values (2 SD below the mean), and a 33 % lower CTRA gene expression level for those with relatively high vs low RMSSD HRV values. Broadly similar findings emerged from secondary unadjusted analyses that did not control for demographic factors and health behaviors (CTRA association with SDNN: −0.0014 ± 0.0008, *p* = 0.113; RMSSD: −0.0010 ± 0.0005, *p* = 0.088), but these results only trended toward significance due to failure to account for the confounding effects of demographic factors and health behaviors which both affect CTRA gene expression and correlate with HRV (as noted above).

HRV was most strongly associated with the CTRA profile overall, as no significant associations emerged in separate secondary analyses of inflammatory or Type I interferon gene subcomponents of the CTRA (both *p* > 0.05). However, consistent with previous research (Rahal et al., 2021; Sloan and Cole, 2021), the Type I interferon subcomponent did show a nonsignificant trend toward positive association with HRV in covariate-adjusted analyses (p = 0.097 and 0.078 for SDNN and RMSSD, respectively) whereas inflammatory gene expression showed no such trend for either HRV measure (p = 0.668 and 0.717, respectively).

**Fig. 2:**
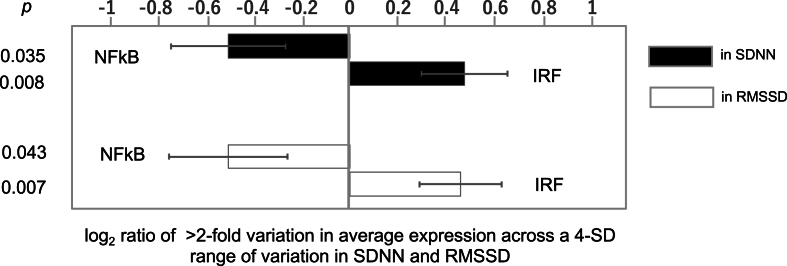
Activity of NF-kB and IRF transcription factors in HRV-related differences in genome-wide transcriptional profiles SDNN: standard deviation of NN intervals; RMSSD: root mean square of successive differences; IRF: interferon response factor.

To provide convergent validation of HRV association with CTRA gene expression using an alternative analytic approach, we used TELiS promoter-based bioinformatics analysis to quantify the activity of NF-κB and IRF transcription factors in HRV-related differences in genome-wide transcriptional profiles (Fig. 2). Among 1409 genes showing >2-fold variation in average expression level across a 4-SD range of variation in SDNN (i.e., ranging from a Z score of −2 to + 2; 1156 up-regulated and 253 down-regulated), results linked HRV to significantly up-regulated IRF activity (1.39-fold ratio of IRF TFBMs in genes up-regulated in high vs. low SDNN; log_2_ ratio = 0.474 ± 0.177 SE, *p* = 0.008, FDR *q* = 0.008) and significantly down-regulated NF-κB activity (0.71-fold, −0.500 ± 0.235, *p* = 0.035, FDR *q* = 0.043). In parallel analyses of 1516 genes showing >2-fold variation in average expression across a 4-SD range of variation in RMSSD (1263 up-regulated and 253 down-regulated) results linked HRV to significantly up-regulated IRF activity (1.37-fold, 0.457 ± 0.169, *p* = 0.007, *q* = 0.008) and significantly down-regulated NF-κB activity (0.71-fold, −0.500 ± 0.245, *p* = 0.043, *q* = 0.043).

## 4 Discussion

These results identify a significant inverse association between HRV and CTRA gene expression in a sample of young adults in Japan. These findings extend the existing literature in western countries (Rahal et al., 2021; Sloan and Cole, 2021) by linking PNS activity to reduced CTRA gene expression in a sample of young adults from Japan, including in analyses that controlled for ethnicity. In other words, the benefits of higher HRV in relation to CTRA generalizes to a population of young adults in Japan. This finding is notable in light of several previous studies that have documented distinct risk factors for inflammatory biology in Asian vs Western populations. The present data suggest that basic PNS neuro-immune regulation of the CTRA appears similar in Japanese individuals, as part of broader East Asian region, and in Western populations, and thus any differences in more distal risk factor-health relationships would likely reflect cross-cultural differences in the specific upstream psychological pathways leading to PNS activation (e.g., differences culturally-mediated stress or coping) rather than the downstream neuro-immune interactions the proximally regulate gene expression in circulating immune cells.

Both HRV and CTRA are closely associated with psychological states. For instance, psychologically healthy individuals (i.e., those with higher well-being and lower stress experience) tend to show higher HRV and lower CTRA (e.g., Cannard et al., 2024; Fredrickson et al., 2015; Sloan et al., 2016). Indeed, the body utilizes three biological systems — autonomic nervous system (as reflected by HRV), the immune system (as reflected by such as CTRA) and the endocrine system — that communicate with each other to maintain homeostasis against a stress Cole, 2019, Janig, 2022, Zefferino et al., 2021. On the other hand, chronic stress exposures lead to higher SNS activity and augmented CTRA, both of which contribute to immune-mediated diseases. Consistent with this overall homeostatic perspective, the present results link higher HRV to reduced levels of pro-inflammatory signaling through the NF-κB pathway and increased activity of the IRF transcription control pathway involved in innate antiviral response. The convergence between these measures of genome-wide transcription factor activity and the more focused analyses of pre-specified CTRA composite scores provides an internally consistent portrait of PNS down-regulation of the CTRA gene regulatory program (Cole, 2019).

Some previous studies have suggested that race and ethnicity could affect the relationship between HRV and plasma inflammatory markers such as IL-6 (Hill and Thayer, 2019; Park and Thayer, 2014). Generally, lower HRV tends to relate to higher risk for cardiovascular disease, but Hill and Thayer (2019) found that African Americans tend to have higher HRV, compared to European Americans, while also showing higher cardiovascular risk compared to European counterparts. Additionally, African American women have been reported to show elevated levels of inflammatory cytokines, such as IL-6 and C-reactive protein (CRP), compared to their European American counterparts (Park and Thayer, 2014). Rahal et al. (2021) investigated the association between HRV and Type I interferon gene expression, and their supplementary analysis of six ethnic groups showed significantly lower CTRA gene expression in Asian American youths relative to Caucasian youths. However, they did not test whether the strength of association between HRV and CTRA activity was different across the two groups (perhaps because there were only n = 9 Asian American participants in their study). In a large-scale comparative study of elderly populations in the U.S. and Japan, Miyamoto et al. (2019) identified population differences in the specific psychological factors that related to health outcomes (e.g., self-reported health, number of chronic disease and mental disorder). In the U.S., independence (operationalized as high personal control) was strongly associated with health, while in Japan, interdependence (high relational harmony and low relational strain) was more strongly linked to health. This suggests that the psychological and social factors contributing to better health may vary across cultures. Additionally, Lee et al. (2023) demonstrated that Japanese undergraduates tend to perceive greater stress from the interpersonal situation than European-Canadian undergraduates, which the investigators attributed to differences in cultural backgrounds (i.e., individualism vs. collectivism). However, neither of these studies directly measured the activity of the mediating neurobiological pathways (e.g., PNS autonomic activity as indexed by HRV), so it remains unclear whether the cross-cultural differences identified there reflect differences in psychological or social activation of neurobiology, or if they reflect differences in neurobiological regulation of immune system biology. Collectively, these observations underscore the significance of our investigation in elucidating the extent to which neuro-immune interaction can be generalized across cultural and ethnic backgrounds.

To determine whether population based in Japan, as a representative East Asian group showed similar or different autonomic-immune regulation dynamics to previously studied Western populations, we investigated whether PNS activity showed the same inverse association with CTRA gene regulation as previously documented in US study samples. We found that the inverse relationship between HRV and CTRA gene expression observed in previous Western samples is also observed in Japan. As in previous research (Rahal et al., 2021; Sloan and Cole, 2021), we also found that the overall CTRA effect most strongly involved a positive association between HRV and the Type I interferon subcomponent of the CTRA composite (although this effect did not reach statistical significance in this sample, likely due to the limited sample size available in this study), whereas no trend toward association was observed for the inflammatory subcomponent of the CTRA. However, the present study's genome-wide bioinformatics analysis linking HRV to up-regulated IRF transcription factor activity provides convergent validation for PNS support of innate antiviral response. Overall, these results suggest that cross-cultural differences in relationships between psychosocial factors and immune function are more likely to reflect differences in social or cultural processes that stimulate autonomic physiology (e.g., via differential cultural value system) rather than cross-cultural differences in the more basic physiological processes that mediate neuro-immune regulation.

However, these conclusions are limited in scope by the fact that we studied only one aspect of neuro-immune regulation (i.e., parasympathetic interactions with CTRA gene expression) in a small sample from a single population (i.e., Japanese adults). Whether similar cross-cultural consistencies occur for other aspects of neuroimmune regulation or for other East Asian populations remains to be explored in future research. Moreover, there are some psychological domains where East Asian and Western samples show similar associations with CTRA gene expression (e.g., reduced CTRA gene expression with greater eudaimonic well-being, as the association between CTRA and psychological well-being has been found not only in Western countries but also in Japan and Korea) (Kitayama et al., 2016; Lee et al., 2020). The present conclusions are also limited by the small sample size available in this study, which reduces statistical power and may undermine this study's ability to detect some effects that have previously been observed in other larger samples (e.g., significant positive associations between HRV and the Type I interferon subcomponent of the CTRA indicator gene set) (Rahal et al., 2021; Sloan and Cole, 2021).

In addition to the limited scope of conclusions that can be drawn from this study's specific sample, other limitations of this analysis include its cross-sectional nature (which precludes any conclusion regarding the causal relationship between HRV and CTRA gene expression), and an a priori focus on CTRA gene expression (i.e., other gene transcriptional correlates of HRV may well exist, but were not identified here due to the limited sample size which precludes any well powered genome-wide exploratory/discovery analysis at the level of individual genes; such analyses are an important topic for future research in larger samples). The health significance of these transcriptomic differences also remains to be determined in future research. To confirm the reproducibility of these findings, future research should aim to increase sample sizes, and experimental manipulation of HRV will likely contribute to elucidating the causal relationship between HRV and CTRA. For example, psychological or physiological interventions such as mindfulness-based training and exercise have been shown to enhance HRV (e.g., Casanova-Lizón et al., 2022; Su et al., 2024; Tung and Hsieh, 2019; Lindsay et al., 2024) and/or reduce CTRA (Antoni et al., 2016; Black et al., 2019; Creswell et al., 2012).

## 5 Conclusion

Two time-domain measures of HRV (SDNN and RMSSD) were inversely associated with CTRA gene expression in young healthy adults in Japan. These effects are associated with increased activity of the IRF transcription control pathway and decreased activity of NF-κB. Results are similar to those from Western populations, and suggest that PNS regulation of the CTRA is broadly similar in Asian and Western populations, even though the social and psychological processes that activate autonomic activity may differ by culture.

## CRediT authorship contribution statement

**Yoshino Murakami:** Writing – original draft, Visualization, Project administration, Methodology, Investigation, Data curation, Conceptualization. **Takeshi Hashimoto:** Writing – review & editing, Supervision, Resources, Project administration, Methodology, Funding acquisition, Conceptualization. **Steve Cole:** Writing – review & editing, Software, Resources, Methodology, Formal analysis, Conceptualization.

## Funding

This research was supported by Grant-in-Aid for Scientific Research from the Japanese Ministry of Education, Culture, Sports, Science, and Technology (to T.H.), grant numbers #21H03384.

## Declaration of competing interest

The authors declare the following financial and personal relationships that could be viewed as potential competing interests: Takeshi Hashimoto received financial support from the Grant-in-Aid for Scientific Research (Grant Numbers: 21H03384) provided by the Japanese Ministry of Education, Culture, Sports, Science, and Technology.

All other authors declare that they have no known competing financial interests or personal relationships that could have appeared to influence the work reported in this paper.

## Data Availability

Data will be made available on request.
